# Electromagnetic Interference (EMI) Shielding and Thermal Management of Sandwich-Structured Carbon Fiber-Reinforced Composite (CFRC) for Electric Vehicle Battery Casings

**DOI:** 10.3390/polym16162291

**Published:** 2024-08-14

**Authors:** Shi Hu, Dan Wang, Josef Večerník, Dana Křemenáková, Jiří Militký

**Affiliations:** 1 Department of Material Engineering, Faculty of Textile Engineering, Technical University of Liberec, Studenska, 1402/2, 461 17 Liberec, Czech Republic; shi.hu@tul.cz (S.H.); dana.kremenakova@tul.cz (D.K.); jiri.militky@tul.cz (J.M.); 2 Vecernik s.r.o, Alšovice 54, 468 21 Pěnčín, Czech Republic; jvecernik@seznam.cz

**Keywords:** electrical vehicle battery casing, carbon fiber-reinforced composites (CFRC), dynamic mechanical analysis, thermal insulation properties, EMI shielding, Joule heating

## Abstract

In response to the growing demand for lightweight yet robust materials in electric vehicle (EV) battery casings, this study introduces an advanced carbon fiber-reinforced composite (CFRC). This novel material is engineered to address critical aspects of EV battery casing requirements, including mechanical strength, electromagnetic interference (EMI) shielding, and thermal management. The research strategically combines carbon composite components with copper-plated polyester non-woven fabric (CFRC/Cu) and melamine foam board (CFRC/Me) into a sandwich-structure composite plus a series of composites with graphite particle-integrated matrix resin (CFRC+Gr). Dynamic mechanical analysis (DMA) revealed that the inclusion of copper-plated fabric significantly enhanced the stiffness, and the specific tensile strength of the new composites reached 346.8 MPa/(g/cm^3^), which was higher than that of other metal materials used for EV battery casings. The new developed composites had excellent EMI shielding properties, with the highest shielding effectives of 88.27 dB from 30 MHz to 3 GHz. Furthermore, after integrating the graphite particles, the peak temperature of all composites via Joule heating was increased. The CFRC+Gr/Me reached 68.3 °C under a 5 V DC power supply after 180 s. This research presents a comprehensive and innovative approach that adeptly balances mechanical, electromagnetic, and thermal requirements for EV battery casings.

## 1. Introduction

In recent years, the automotive industry has witnessed a remarkable surge in the development of electric vehicles (E-vehicles or EVs), propelled by the rapid advancements in battery technology [1,2,3,4]. As the quest for more sustainable transportation solutions gains momentum, researchers and engineers are focusing on enhancing various aspects of EV design to optimize performance, efficiency, and environmental impact [5,6,7]. One critical area of exploration is the materials used for the exterior casing of electric vehicle batteries. Common choices include aluminum alloys, which offer light weight and excellent thermal conductivity, contributing to overall vehicle weight reduction and improved energy efficiency [8,9]. Nickel–cobalt–manganese (NCM) alloys, originally employed as positive-electrode materials in lithium-ion batteries, find application in battery casings due to their high energy density and chemical stability [10]. Additionally, thermoplastic engineering polymers like polypropylene (PP) and polyamide (PA) are utilized for their lightweight attributes, chemical resistance, and cost-effectiveness [11,12]. Although sacrificing some strength compared to that of metals, these plastics are suitable for certain applications. Carbon fiber composite materials (CFRC), which comprise carbon fibers and resins, demonstrate superior strength and lightweight characteristics, contributing to weight reduction and enhanced efficiency, which make them emerge as a promising candidate. The appeal of carbon fiber composites lies in their inherent superior properties, such as high strength-to-weight ratio, corrosion resistance, and exceptional durability [13,14,15,16]. These characteristics make carbon fiber composites particularly attractive for applications demanding lightweight yet robust materials [17,18,19,20]. As the automotive industry increasingly shifts towards electrification, the need for materials capable of meeting the specific challenges posed by electric vehicle battery casings becomes paramount.

Despite the increased interest in electric vehicles, there remains a notable dearth of comprehensive research specifically addressing the potential of carbon fiber composite materials for battery casings. Many existing studies focus on performance in isolation, overlooking the intricate interplay of mechanical, electromagnetic, and thermal factors. M. S. Santhosh [21] studied the possibilities of using an E-Glass/phenolic matrix/APP laminate composite as an E-vehicle battery casing. The related mechanical tests, including tensile, flexural, low-velocity impact, and quasi-static indentation tests, were conducted; however, electromagnetic shielding and thermal properties’ tests are missing in that study. Monu Malik applied the phase change material (PCM) as one possible material for an E-vehicle battery casing to enhance the thermal management of the battery. The thermal conductivity of the PCM with a mixed graphite material reached 70 W·m^−1^·K^−1^. Still, the mechanical and electromagnetic shielding properties were not discussed in that paper [22].

Contrasted with traditional metallic materials typically employed in electric vehicle exteriors, carbon fiber composites face a unique set of challenges that necessitate meticulous consideration. One of the primary concerns is whether these composite materials can meet the stringent mechanical performance requirements expected of automotive components. Given their renowned mechanical prowess, carbon fiber composites are poised to fulfill these demands. Another critical consideration is the electromagnetic shielding effectiveness of carbon fiber composite materials. Electric vehicles, particularly during battery operation, generate substantial electromagnetic fields [23,24,25]. The protective shield provided by metallic battery cases in conventional vehicles ensures a minimal impact on human health. However, as non-metallic materials, many newly developed carbon fiber composites overlook the crucial assessment of their electromagnetic shielding effectiveness, a pivotal factor in ensuring the safety and performance of electric vehicle batteries. Thermal management poses yet another challenge for carbon fiber composite materials intended for electric vehicle battery casings. The operational heat generated by batteries necessitates efficient heat dissipation to maintain optimal performance and extend battery lifespan [26,27]. Conversely, in colder environments, the composite material must provide adequate insulation to counteract the adverse effects of low temperatures on the electric vehicle’s range and overall performance [28,29].

This paper introduces a novel approach to address these challenges, presenting a carbon fiber composite material that undergoes water-soluble resin processing to achieve uniformity in resin distribution. To enhance electromagnetic shielding effectiveness and ensure optimal thermal conductivity, copper-plated polyester non-woven fabric was strategically integrated into the composite structure, and a graphite particles-mixed resin matrix was used for another group of samples for comparison. Additionally, to meet insulation requirements, melamine foam boards were incorporated between two layers of carbon fiber composite material, effectively improving thermal resistance. Mechanical properties were meticulously characterized through a DMA three-point bending test, demonstrating the material’s suitability for automotive applications. The culmination of these innovations presents a carbon fiber sandwich structure with the potential to meet and exceed the diverse performance requirements for electric vehicle battery casings. This research bridges critical gaps in the current understanding of carbon fiber composite materials in the context of electric vehicle battery casings. By addressing mechanical, electromagnetic, and thermal considerations, our approach offers a modular performance design solution that could pave the way for the widespread adoption of carbon fiber composites in the rapidly evolving landscape of electric vehicle battery casings.

## 2. Experiment

### 2.1. Materials

The carbon fabric with 245 g/m^2^ was supplied from the Havel composite company, Czech Republic. The central layer was polyester nonwovens with 30 g/m^2^ plated with copper particles supplied from Bochemie company, Czech Republic, under the name Meftex 30^®^. The epoxy resin CHS Epoxy 200 used in the composite processing were from Havel composite company, Czech Republic. The melamine foam was supplied from BASF, Germany. The commercial name of the melamine foam is Basotect^®^. The graphite particles and other related chemicals were purchased from Sigma-Aldrich. The basic physical characteristics of the greige samples are presented in Table 1.

### 2.2. Fabrication of the Composites

The hybrid composites were manufactured by a hot press molding procedure. Regarding the composite matrix system, there were two kinds of matrix prepared for this research. The matrix of an epoxy resin mixed with 10% of the catalyst KSCN was prepared first. In order to enhance the electrical conductivity of the matrix, 10 wt% graphite particles were mixed with the matrix system via magnetic stirring for 30 min. In order to compare the different properties of the composites, the weight fraction of the CF cloth in all CFRCs was controlled at about 60 wt%.

**Figure 1:**
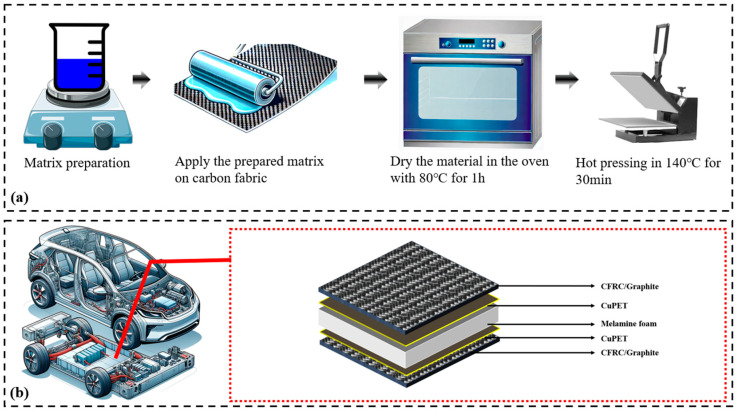
(**a**) Carbon fiber-reinforced composite (CFRC) fabrication process (**b**) The multilayer sandwich structure of the CFRC+Gr/Cu/Me.

The prepared matrix was applied onto the arranged layers of CF via the hand lamination process. By using a hand-rolling tool, the extra resin was pressed out of the CF. The prepared semi-composites were placed in room temperature for 24 h in order to stabilize and to evaporate the water. Then, the samples were put into an oven at 80 °C for 1 h to start the curing process. Lastly, the semi-product was arranged and pressed in a heated hand presser at a temperature of 140 °C for 30 min. In the sample with graphite particles, the particles were first mixed with the resin matrix by magnetic stirring for 30 min with good distribution, then the prepared matrix system was applied onto a single layer by the same procedure as in other samples. The detailed fabrication process is presented in Figure 1, and the basic physical characteristics of the CFRC are presented in Table 2.

## 3. Methods

### 3.1. Density

Density is one of the most important factors in determining the material properties of polymer composites and is defined as mass of the material per unit of volume, measured as per the standard ASTM D1895 [30]. The mass was measured via analytical balance, and the thickness of the sample was measured using a thickness meter Thickness Gauge D-2000 with a 50 mm presser foot diameter. The pressure applied during the measurement was set at 1.0 kPa. The measurements were done five times, and the arithmetic mean and 95% confidence interval were calculated.

### 3.2. EMI Shielding

The EMI shielding effectiveness test was performed using the coaxial transmission line method according to ASTM 4935-10 [31] to evaluate flat materials. This method presumes a plane wave impact on a shielding material in the frequency band from 30 MHz to 3 GHz. This measuring equipment consists of a coaxial specimen holder (supplied by Electro-Metrics, Inc. (New York, NY, USA), EM-2107A). The input and output signals were transferred to a ZNC3 vector network analyzer from Rohde & Schwarz (Munich, Germany). The analyzer was designed to generate and receive electromagnetic signals. The power ratio can calculate the SE (forward transmission coefficient S21) without and with the shielding material. The calculation is shown in Equation (1):

(1) $$SE\left(S21\right)=-10log\frac{P_{1}}{P_{2}}=10log\frac{P_{2}}{P_{1}}$$

P_1_ is the received power without the shielding material, and P_2_ is the received power with the shielding material present. The electromagnetic wave reflection coefficient SE_R_ (S11) interpreted that the electromagnetic wave signal from the transmitting antenna was reflected by the shielding material and received by port 1. The ratio of the receiving reflected electromagnetic wave powers P_3_ and P_1_ shows the input reflection coefficient as in Equation (2):

(2) $${SE}_{R}(S11)=10log\frac{P_{3}}{P_{1}}$$

According to the transmission line theory, total SE_T_ can be interpreted by following Equations (3) and (4):

(3) $$SE_{T}={SE}_{A}+{SE}_{R}+{SE}_{M}$$

(4) $$1=T_{EM}+A_{EM}+R_{EM}$$

In Equation (3), SE_T_ is shielding effectiveness, SE_R_ is reflection loss, SE_A_ is absorption loss, and SE_M_ is multi-reflection loss. Usually, the factor SE_M_ can be mathematically positive or negative (in practice, it is always negative).

In Equation (4), the coefficient of transmittance (T_EM_) and reflectance (R_EM_) can be calculated by Equations (5) and (6):

(5) $$SE_{T}=-10\log_{10}T_{EM}$$

(6) $${SE}_{R}=-10\log_{10}(1-R_{EM})$$

### 3.3. Thermal Properties

According to the standard ISO 11092 [32], Alambeta was the thermal properties measuring equipment used in this work, and thermal conductivity and thermal resistance were selected. Thermal resistance is the value obtained by dividing the temperature difference between any two parts by the heat flux flowing between the two points. The sample was set on the bottom measuring plate during the test, and the head plate went down and touched the sample with 200 Pa of pressure. Then, the heat flow value of the sample was analyzed and calculated, and the final thermal resistance and conductivity values were displayed. The measurement ambient temperature was 24 °C, and the relative humidity was around 40%. Every sample was tested five times at different points, and the average value was calculated.

### 3.4. DMA 3-Points Bending Testing

The DMA was conducted on a METTLER TOLEDO (Prague, Czech Republic) machine in a 3-points bending mode. The sample was prepared with dimensions of approximately 40 mm in length and 15 mm in width. Considering the thermal stability of carbon composites and melamine foam, the temperature ranged from 25 to 250 °C and from 25 to 125 °C for melamine foam-integrated samples (CFRC/Me, CFRC+Gr/Me, CFRC/Cu/Me, CFRC+Gr/Cu/Me). The heating rate was set at 3 °C/min. The frequency was set to 1 Hz. In the 3-points bending mode, the sample was placed into a fixed holder. For each test, the length, width, and thickness of the sample were measured and entered into the control system. Each sample within a given group underwent the DMA bending mode procedure three times, and the resultant values were averaged to calculate parameters such as storage modulus, loss modulus, and loss factor (tan δ).

### 3.5. Tensile Strength Test

In this study, the tensile properties of composite samples were assessed using a universal testing machine (TIRA test 2300, Berlin, Germany) in compliance with the ASTM D-3039 testing standard [33]. Each sample about 25 mm wide and 150 mm long was subjected to the tensile test procedure five times, and the resulting values were averaged to determine parameters such as stress and strain.

### 3.6. Joule Heating Test

**Figure 2:**
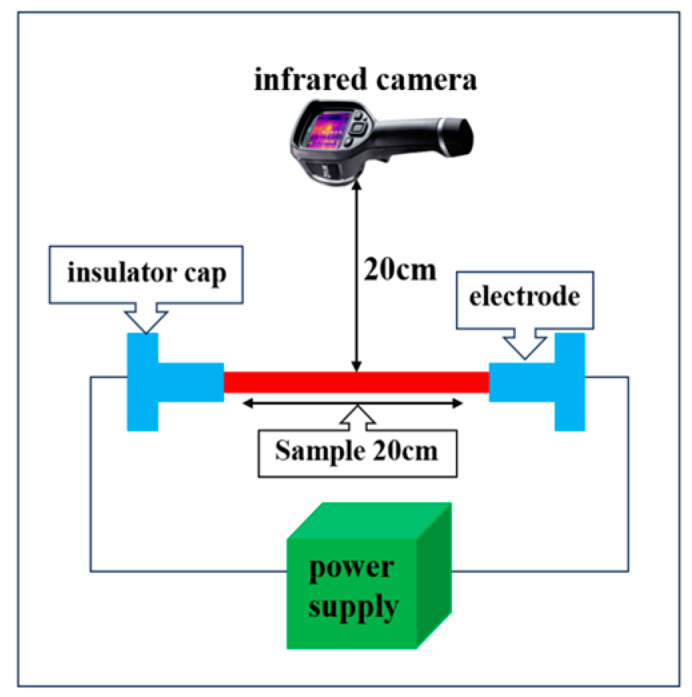
Joule heating test setup.

The Joule heating effect in composite samples was investigated using an infrared camera (FLIR-E6390, FLIR, Malmö, Sweden) and an electrical power supply (TIPA PS3010, TIPA EU, Czech Republic). The composite sample’s two edges were connected to the power supply through a voltage regulator, with the electrodes spaced 20 cm apart. The infrared camera was positioned 20 cm above the sample surface to monitor temperature changes. An electric current was applied at different voltages of 2.5, 5, and 7 V, and the temperature at the midpoint between the two electrodes was recorded for 5 min. For all measured composite samples without melamine foam, the surface temperature stabilized after approximately 120 s. For samples with melamine foam, the surface temperature stabilized after about 180 s. At these respective time points, 120 s for samples without melamine foam and 180 s for samples with melamine foam, the electricity was cut off to observe the heat-release process. Figure 2 illustrates the Joule heating of the composite samples.

## 4. Results and Discussion

### 4.1. Basic Physical and Morphology Characterization

**Figure 3:**
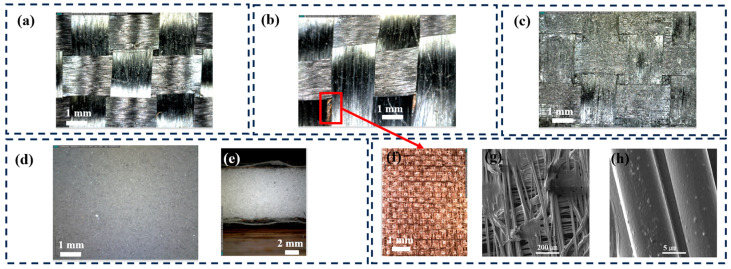
Carbon fiber-reinforced composite surface morphology: (**a**) CFRC surface, (**b**) CFRC/Cu surface, (**c**) CFRC+Gr/Cu surface, (**d**) melamine foam surface structure, (**e**) CFRC/Me cross-section structure, (**f**) copper-coated PET nonwovens layer, (**g**) SEM picture of CuPET with fiber distribution, (**h**) Cu particle distribution on PET fibers of CuPET.

The basic physical and morphological characteristics of the composite materials are detailed in Table 2 and Figure 3. Following the fabrication process, an even distribution of the epoxy matrix was observed on the fiber surfaces, as depicted in Figure 3a–c. The composite CFRC/Cu was designed as a sandwich structure, characterized by a central layer of a copper-coated PET fabric (CuPET), visible through the apertures between the carbon yarns (Figure 3b). The structure of CuPET, displaying a typical nonwoven fabric configuration, is illustrated in Figure 3f. Employing a thermal bending method, horizontal and vertical filaments were produced as nonwoven structured fabrics. The coated Cu particles on the PET filament were also evident in the SEM images (Figure 3h).

The graphite-integrated CFRC samples (CFRC+Gr) had physical characteristics similar to those of normal resin-reinforced samples. However, judging by the microscope picture, the surface roughness was obviously increased compare to that of no-particles-mixed samples (Figure 3c). Due to the limited amount of mixed graphite particles, the change in density of the CFRC+Gr group samples was not significant compared to that of the no-particles-mixed samples.

Regarding the hot-pressing procedure of the CFRC series, it resulted in a relatively thin composite material, with the exception of the CFRC/Me variant. The thickness of CFRC/Me was notably higher compared to that of the other samples, attributable to the substantial thickness of the melamine foam (Figure 3d,e). Moreover, the thicker and porous structure of CFRC/Me reduced its density compared to that of other materials. Density measurements of the composites revealed that they all possessed densities lower than 1 g/cm^3^, indicating a density lower than that of water. 

In contrast, aluminum alloys, commonly utilized in EV battery casings, have densities ranging from 2.64 to 2.8 g/cm^3^, significantly higher than that of the CFRC materials. The low density of the developed composites underscores their potential advantage for application in EV battery casings.

### 4.2. Mechanical Properties

**Figure 4:**
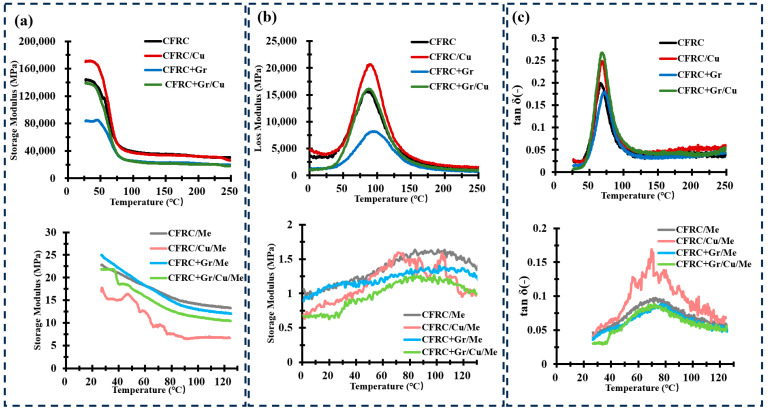
DMA 3-points bending test results of (**a**) storage modulus, (**b**) loss modulus, and (**c**) loss factor (tan δ).

Considering the actual working situation of the composite application in EV battery casings, the material mostly faces impact deformation under vibration and dynamic changed temperature. In this case, the mechanical properties of the composites were tested with the DMA 3-points bending testing. Considering the heat stability of the melamine foam for the risk of damaging the DMA equipment, the maximum temperature for melamine foam-integrated composites was set to 120 °C. The DMA 3-points bending test results are shown in Figure 4.

The storage modulus primarily reflects the stiffness variation of the material at different temperatures under a constant vibration frequency. The results of the storage modulus are shown in Figure 4a. For the composite materials without melamine foam, the stiffness of the composites showed little change at temperatures below 55 °C. The stiffness of all materials began to decrease rapidly above 55 °C, and the rate of decrease slowed down after 75 °C, stabilizing around 150 °C. In particular, regarding the CFRC/Cu sample, its initial storage modulus was higher than that of CFRC, approximately 180,000 MPa. It is evident that the integration of CuPET enhanced the overall stiffness of the material. When graphite (Gr) was added to CFRC(CFRC+Gr), the initial storage modulus was about 120,000 MPa, significantly lower than that of CFRC. The addition of graphite seemed to reduce the initial stiffness. This could have been due to the possibly weaker interfacial bonding between graphite and the resin matrix compared to that in CuPET. Such weak interfacial bonding may lead to relative sliding or separation between graphite particles and the resin matrix under stress, thereby reducing the stiffness of the material. Considering the dispersibility of graphite, if graphite particles are not uniformly dispersed in the resin, they are prone to forming agglomerates, which can become stress concentration points, weakening the overall mechanical properties of the material and leading to a decrease in stiffness. The addition of a melamine foam board significantly reduced the initial stiffness of the carbon fiber composite material. The stiffness of the sample gradually decreased over the entire temperature range, remaining relatively stable at around 110 °C. The inclusion of graphite particles in the resin slightly increased the initial modulus, but the impact was minimal.

The loss modulus represents the material’s ability to dissipate energy in the form of heat or molecular rearrangement during deformation (Figure 4b). Overall, for composites without the addition of melamine foam, the peak value of the loss modulus was far higher compared to that of composites with foam. This is mainly because the resin matrix in composites without foam exhibited significant internal friction near the glass transition temperature (T_g_). In contrast, the inclusion of melamine foam reduced the overall internal friction level due to the foam’s low internal friction characteristics, stress distribution effects from its porous structure, and interfacial effects. Among these, the composite with added CuPET showed the highest peak value. This suggests that the addition of copper-plated fabric resulted in greater internal friction and viscoelastic loss at higher temperatures. Conversely, the inclusion of graphite particles lowered the peak value of the loss modulus.

For composites with melamine foam, the loss modulus showed considerable fluctuation, and the peak became less distinct compared to that of composites without foam. This may be due to the introduction of a porous and heterogeneous phase structure by the melamine foam. The porosity and heterogeneity of the foam caused variations in performance across different regions of the composite. In DMA testing, such non-uniformity led to fluctuations in the measured loss modulus. Additionally, the interface between the foam and the resin matrix was likely more complex. The adhesive properties, bonding strength, and stress transfer characteristics at the interface may not be as stable as in homogeneous matrix materials. During thermal processing, differences in thermal expansion coefficients and stress concentration in the interfacial regions can cause fluctuations in the loss modulus. These interfacial effects result in the internal friction behavior near T_g_ being less concentrated into a single peak, instead presenting a more dispersed pattern.

The value of the loss factor tanδ usually indicates the relative viscosity and elastic behavior of the material, and the temperature corresponding to the peak of the tanδ curve is usually considered to be the glass transition temperature (T_g_). For all composites, the peak corresponding to T_g_ was also basically in the range of about 70 °C, since the choice of resin was unique (Figure 4c). Among them, the introduction of a copper-coated polyester fabric (CFRC/Cu) raised the peak value of the loss factor, indicating higher internal friction and energy absorption in this temperature range.

To compare the developed composite material with commonly used metal material for EV battery casings, the tensile test was also applied. The results are presented in Table 3.

For CFRCs without the incorporation of melamine foam, the tensile strength ranged from 167.6 to 295 MPa, and the tensile modulus ranged from 2.7 to 5.5 GPa. It was evident that the inclusion of graphite particles significantly enhanced the tensile properties. Comparing CFRC with CFRC+Gr and CFRC/Cu with CFRC+Gr/Cu, the tensile strength increased by 12.2% and 49.3%, respectively. According to published research, this improvement can be attributed to the addition of graphite particles to the resin matrix, which enhances the interfacial bonding between the carbon fibers and the resin matrix. Strong interfacial bonding facilitates efficient stress transfer, thereby enhancing the overall strength of the composite material. Upon integrating melamine foam, the cross-section increased significantly. However, the mechanical strength of melamine foam is substantially lower than that of carbon composites. Consequently, the tensile strength of melamine foam-integrated composites was considerably lower than that of pure CFRC or CFRC+Gr.

Compared to typical metals used for EV battery casings, such as 6061-T6 aluminum alloy, 7075 high-strength aluminum alloy, high-strength steel (HSS), and ultra-high-strength steel, the CFRCs examined in this study exhibited a certain degree of comparability in tensile strength. Notably, the CFRC+Gr/Cu sample demonstrated a tensile strength of 295 ± 32.2 MPa, which is comparable to the referenced tensile strength of 6061-T6 aluminum alloy, approximately 310–350 MPa. Although carbon fiber composites have a significant gap in tensile strength compared to that of the other three materials used for electric vehicle battery casings, they offer a high specific modulus and specific strength. Specific modulus is the ratio of modulus to density, and specific strength is the ratio of strength to density.

Generally, for a given structural stiffness and strength, a material with a higher specific modulus and specific strength will result in a lighter structure. As seen in Table 3, the specific strength of metal materials ranges from 114.8 to 224.4 MPa/(g/cm^3^), while that of the carbon fiber composites ranged from 128.2 to 346.8 MPa/(g/cm^3^). Overall, carbon fiber-reinforced composites have higher specific strength values than steel and aluminum alloys do. This is a key reason why using composites in the design of electric vehicle battery casings can significantly reduce weight.

Carbon fiber composites also possess unique characteristics in electromagnetic shielding and thermal management, making them suitable for the development of electric vehicle battery casings. These aspects will be discussed in detail in the following sections.

### 4.3. EMI Shielding Performance

In the majority of electric vehicles, electromagnetic field (EMF) strength within the cabin is considered safe for both the driver and passengers. This safety is partly attributed to the metal shell of the battery and the use of metallic materials in the battery casing, which effectively shield from additional electromagnetic radiation [38]. Consequently, when substituting the metal material of the battery casing with a carbon composite, it is imperative that the EMI shielding efficacy of the carbon composite material is at least equivalent to that of metal materials. This substitution is crucial to maintain a secure electromagnetic field environment within the vehicle. Therefore, the EMI shielding performance of the composite material was rigorously evaluated.

**Figure 5:**
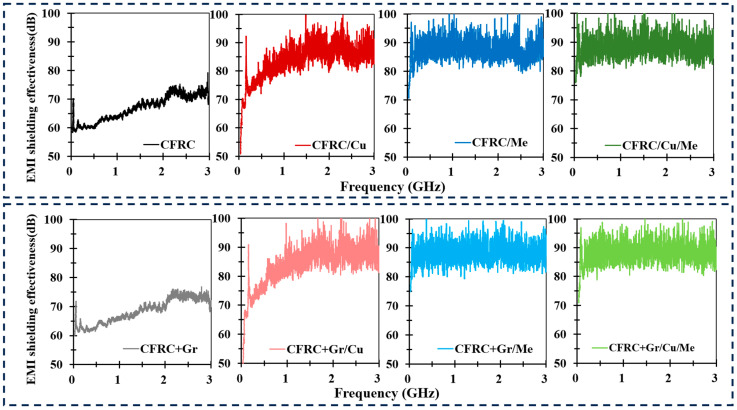
EMI shielding performance of carbon composite materials: CFRC, CFRC/Cu, CFRC/Me, CFRC/CU/Me, CFRC+Gr, CFRC+GR/Cu, CFRC+Gr/Me, and CFRC+Gr/Cu/Me.

Electric vehicle batteries are sources of electromagnetic waves, primarily emanating from their charging and discharging processes and associated electrical systems. The spectrum of electromagnetic waves produced by electric vehicles spans a broad range, encompassing both low-frequency and high-frequency components [39,40]. The low-frequency segment is typically linked to battery charging and discharging, motor control, and power conversion, with frequencies varying from tens to several thousand hertz. Conversely, the high-frequency electromagnetic waves are principally associated with the electronic equipment, switching power supplies, and communication systems in electric vehicles. These high-frequency components can range from a few kilohertz to hundreds of megahertz or even higher. To cover the widest frequency band, considering the limitations of the testing equipment, the EMI shielding property was assessed within the frequency range of 30 MHz to 3 GHz. Note that due to exceeding the specified frequency range, the test results above 1.5 GHz may not fully comply with the standard methods or accuracy requirements according to the standard ASTM 4935-10. However, the test equipment, setup, and test environment were fully fulfilling the requirements in the standard. Although the test results for electromagnetic shielding effectiveness above 1.5 GHz may have some accuracy limitations, they still hold significant reference and research value. The results of this assessment are depicted in Figure 5.

The overall EMI shielding capabilities of all conductive samples are depicted in Figure 5. Carbon fabric materials exhibits commendable electrical conductivity, allowing the unprocessed carbon fabric to achieve an EMI shielding efficacy of approximately 60 dB. Following the integration with a resin matrix system, the EMI shielding effectiveness of CFRC remained at a comparable level of around 60 dB. This stability in shielding performance was attributed to the unchanged conductive layer of the carbon fabric post-composite fabrication. The exceptional EMI shielding properties of CuPET have been analyzed and corroborated in our prior studies, demonstrating an EMI shielding performance of approximately 53 dB [41,42,43].

**Figure 6:**
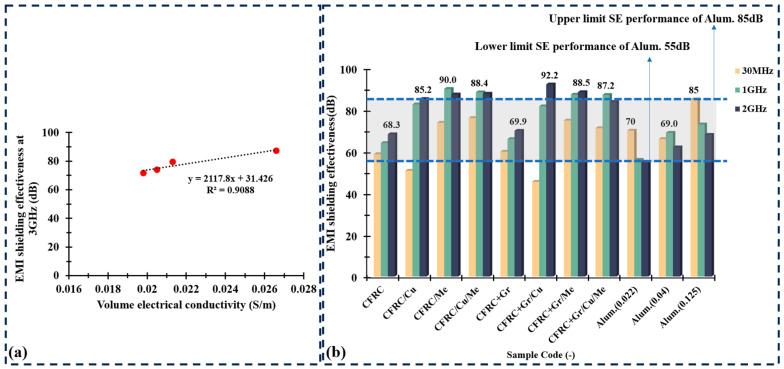
(**a**) The relationship between shielding effectiveness at 3 GHz and volume electrical conductivity. (**b**) The shielding effectiveness value of carbon composites compared to that of aluminum with different thickness.

When two-layer CFRC and one-layer CuPET or melamine foam were configured into a sandwich structure, there was a significant enhancement in the EMI shielding effectiveness of the sandwich-structured composites. Specifically, the CFRC/Cu composite demonstrated an average EMI shielding effectiveness of 83.66 dB across the frequency band from 30 MHz to 3 GHz, which improved 25.1% compared to that of CFRC. The CFRC/Me composite also exhibited superior EMI shielding effectiveness, approximately 87.47 dB. For the complex structured composites with all functional layers CFRC/Cu/Me, the average SE performed as the maximum value of 88.27 dB. After mixing the graphite particles into the matrix, there was only a small enhancement of 1.57 dB in the original CFRCs compared to CFRC+GR, and the SE enhancement in all other samples was not significant. One of the main factors that has a positive influence on the shielding effectiveness is the electrical conductivity of sample. Some research has shown that the SE value can be increased by enhancing the electrical conductivity of the sample. Due to the high electrical resistivity of melamine foam (>10^6^ Ω·m), the electrical conductivity of CFRC/Me and CFRC+Gr/Me was not calculated. Compare CFRC, CFRC/Cu, CFRC+Gr, and CFRC+Gr/Cu samples, the SE value was increased significantly. Considering the electrical conductivity, the SE value at 3 GHz had a linear relationship with the electrical conductivity (Figure 6a).

The analysis of the S21 signal waveform revealed a distinctive zigzag pattern for CFRC(+Gr)/Cu, CFRC(+Gr)/Me, and CFRC(+Gr)/Cu/Me, as illustrated in Figure 5. EMI shielding attenuation is predominantly achieved through three mechanisms: reflection, absorption, and multiple internal reflections within the material. The zigzag waveform observed in the S21 signal can be attributed to electromagnetic wave interference and diffraction, leading to the superposition of wave crests and troughs. This phenomenon is a direct consequence of multiple internal reflections in sandwich materials, where the incorporation of an intermediate layer between conductive materials increases the spacing between them, facilitating multilayer internal reflection.

Figure 6b compares the EMI shielding performance of carbon composites with that of aluminum material. It is well established in related research that the EMI shielding properties of a material are enhanced with increasing material thickness. For aluminum, an increase in thickness from 0.022 mm (Alum.(0.022)) and 0.04 mm (Alum.(0.04)) to 0.125 mm (Alum.(0.125)) results in a rise in EMI shielding effectiveness from 70 to 85 dB at 30 MHz, from 56 to 73 dB at 1 GHz, and from 55 to 68 dB at 2 GHz. Thus, for aluminum materials of varying thicknesses, the EMI shielding effectiveness ranges from dB to 85 dB at frequencies between 30 MHz and 2 GHz. When compared with that of carbon composite materials, the EMI shielding performance of the CFRC/Me sample (73.8 dB–90 dB) was observed to surpass that of aluminum. Similarly, CFRC/Cu/Me (76.1 dB–88.4dB) and CFRC+Gr/Cu/Me (71.2 dB–87.2 dB) demonstrated EMI shielding effectiveness comparable to that aluminum material.

### 4.4. Thermal Conductivity and Resistance

**Figure 7:**
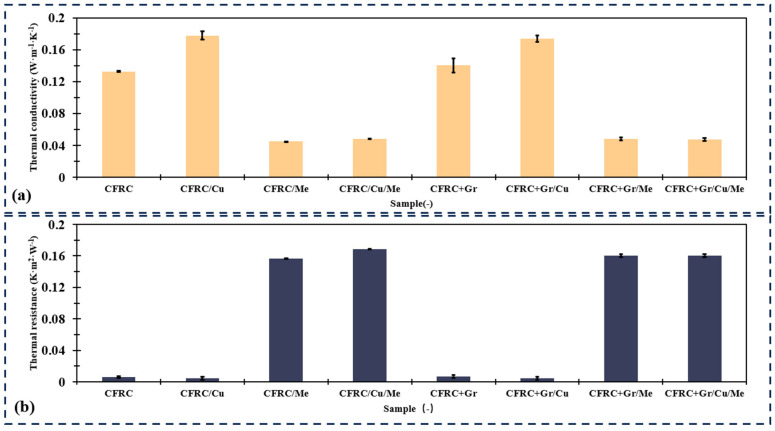
(**a**) Thermal conductivity and (**b**) thermal resistance test of raw and carbon composite materials.

In this analysis, the thermal conductivity properties of eight distinct composite materials were evaluated (Figure 7). The results indicate that CFRC/Cu exhibited the most efficient thermal conductive performance, with a mean thermal conductivity of 0.178 W·m^−1^·K^−1^ and a standard deviation of 0.0056 W·m^−1^·K^−1^. This suggests a high level of consistency and efficiency in its thermal conductive property. After integrating the melamine foam, the composite samples had higher thermal resistivity and lower thermal conductivity. For example, CFRC/Cu/Me demonstrated the highest thermal resistance, with an average value of 0.169 K·m^2^·W^−1^ and a standard deviation of 0.002 K·m^2^ W^−1^, indicating consistent performance despite its higher resistivity. After adding the graphite particles, the changes in thermal conductivity and thermal resistance were not significant. This can be caused by the relatively low concentration of graphite particles and the inhomogenous distribution of the particles.

Samples with melamine foam perform higher thermal resistance, might be more appropriate for insulation or thermal barrier applications. Other materials like CFRC/Cu offer intermediate levels of thermal resistance allowing for selection based on specific application requirements. Considering the application in EV battery casings, the temperature requirement varies in different seasons. The optimal operating temperature range for electric vehicle batteries typically lies between 15 and 35 °C. Within this thermal range, the electrochemical reactions within the battery occur with maximal efficiency, thereby ensuring superior performance and an extended lifespan. EV battery performance significantly diminishes under conditions of extreme heat or cold. Elevated temperatures can precipitate accelerated aging and damage due to the hastened pace of chemical reactions. Conversely, suboptimal low temperatures slow down these reactions, resulting in a reduced effective capacity and diminished power output of the battery. Consequently, numerous electric vehicles are equipped with battery thermal management systems to maintain the battery within an ideal operating temperature range. In this case, samples with melamine foam have good thermal resistance, which could improve the battery’s working temperature in the winter season. However, a cooling system is necessary to maintain the optimal temperature for the EV battery in the summer season.

### 4.5. Joule Heating Material

**Figure 8:**
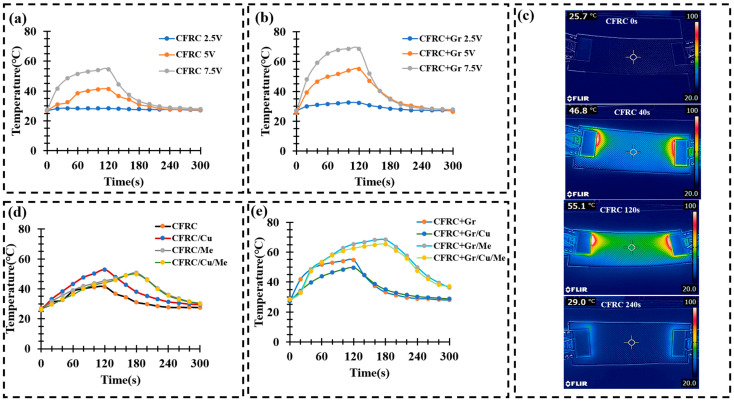
Joule heating test result under different voltages for (**a**) CFRC samples and (**b**) CFRC+Gr samples. (**c**) CFRC temperature changes during the Joule heating (7.5 V) process. (**d**) CFRC series’ and (**e**) CFRC+Gr series’ temperature changes after 300 s under a 5 V direct current.

Considering the application of electric vehicle battery casings, the utilization of conductive materials in colder environmental conditions enables the maintenance of an optimal operating temperature for the battery. This is achieved through the principle of Joule heating, where a specific amount of heat is generated by electrifying the battery casing. Common carbon fiber-reinforced composites (CFRCs), due to the inclusion of an insulating resin, exhibit reduced electrical conductivity and are thus not suitable for generating Joule heat. However, by incorporating conductive graphite particles into the resin, the electrical conductivity of the material can be significantly enhanced, thereby producing higher Joule heat at a relatively reasonable voltage (Figure 8a,b).

The primary results, displayed in Figure 8a,b, clearly indicate an increase in the surface temperature of the CFRC as the voltage increased. At a voltage of 2.5 V, the surface temperature change was not satisfied. When the voltage was increased to 5 V, the surface temperature of the CFRC sample rose to 41.3 °C. CFRC+Gr samples reached 55 °C after 120 s of electrification. When the voltage was increased to 7.5 V, the temperature after 120 s of heating rose to 54.6 °C and 68.5 °C for CFRC and CFRC+Gr samples, respectively. Considering the DMA results, all composites had mechanical property loss around 55 °C, and the T_g_ of all the composites was around 70 °C. It is reasonable to choose a voltage of about 5 V to maintain the optimal working temperature of the EV battery and not over the temperature limit, which would impacting its mechanical properties.

At 5 V, the CFRC material’s temperature increase was stabilizing at around 120 s and reached the peak of about 41 °C. CFRC/Cu showed a faster temperature increase trend, and its peak temperature was 52.8 °C. For samples with melamine foam, the temperature stabilization time was longer (180 s) than in the samples without the foam. After cutting off the electricity, the heat release process of composites with melamine foams was slower than in the samples without foam, which also proved that the thermal resistance of the melamine foam-integrated composites was higher than that of other composites, as shown in Figure 7b. After integrating the graphite particles, the peak temperature of all composites was increased, and especially samples with melamine foam showed good thermal isolation properties. CFRC+Gr/Me reached 68.3 °C, and CFRC+Gr/Cu/Me reached 65.2 °C after 180 s, which was already higher than the temperature with lost mechanical property about 55 °C. In this case, the applied voltage for joule heating should be between 2.5–5 V.

The Joule heating test results presented the good electrical conductivity of the composites, especially after integrating the graphite particles. With the melamine foam, the thermal isolation of the composites was highly improved, which led to the positive influence of the Joule-heated temperature. Considering the application of the EV battery casings in cold weather conditions, the Joule heating composites could be a promising solution for improving the working time of EV batteries by keeping the optimized working temperature. On the other hand, in hot weather conditions, the necessary heat dissipation equipment also needs to be considered.

## 5. Conclusions

This research provides a comprehensive evaluation of sandwich-structured carbon fiber composites designed for potential use in electric vehicle (EV) battery casings. The study investigated the mechanical properties, electromagnetic interference (EMI) shielding effectiveness, thermal conductivity, and Joule heating capabilities of various composite configurations. The inclusion of copper-plated polyester nonwoven fabric (CuPET) within the composite structure significantly enhanced the stiffness and internal friction capacity, as demonstrated by the dynamic mechanical analysis (DMA) results. The addition of graphite particles, while reducing the initial modulus due to weaker interfacial bonding, still contributed to improved electrical conductivity necessary for Joule heating applications. EMI shielding tests showed that composites with CuPET and melamine foam layers achieved superior shielding effectiveness, with the CFRC/Cu/Me configuration demonstrating an average shielding effectiveness of 88.27 dB across the 30 MHz to 3 GHz frequency range. This performance surpasses that of traditional aluminum materials, highlighting the composites’ potential for effective electromagnetic protection in EV battery casings. Thermal conductivity assessments indicated that CFRC/Cu composites exhibited the highest thermal conductivity, making them suitable for applications requiring efficient heat dissipation. Conversely, composites with melamine foam displayed increased thermal resistance, providing excellent insulation properties beneficial for maintaining optimal battery temperatures in colder climates. Joule heating experiments revealed that composites incorporating graphite particles could generate sufficient heat to maintain battery temperatures within the optimal operating range. This capability is particularly valuable for EV battery performance in cold weather conditions, where maintaining warmth is crucial for efficiency and longevity.

In conclusion, the studied sandwich-structured carbon fiber composites exhibited a balanced combination of mechanical strength, effective EMI shielding, adaptable thermal management, and practical Joule heating properties. These attributes position them as promising candidates for EV battery casings, offering a lightweight, efficient, and safe alternative to conventional materials. This research paves the way for further development and potential adoption of carbon fiber composites in the rapidly evolving electric vehicle industry.

## Figures and Tables

**Table 1:** Specifications of carbon fabric, Meftex 30, Basotect, and graphite particles.

Greige Sample	Structure	GSM (g/m^2^)	Thickness (mm)	Density (g/cm^3^)	Supplier
Carbon fabric (CF)	Plain weave carbon	245	0.428	0.53	Havel composite
Meftex 30 (CuPET)	Nonwoven Cu coated PET fabric	34.88	0.135	0.25	Bochemie
Basotect (Melamine)	Melamine foam	68.87	5.94	0.01	BASF
Graphite particle	Particle size < 45 μm	-	-	8.94	Sigma Aldrich

**Table 2:** Carbon fabric-reinforced composites’ basic physical characteristics.

Sample Code.	Description	Structure	GSM (g/m^2^)	Thickness (mm)	Density (g/cm^3^)
CFRC	Carbon fiber-reinforced composite	Two layers	613.15	0.8 ± 0.08	0.76
CFRC/Cu	Carbon composite with Cu-coated PET as the center layer	Sandwich structure	717.89	0.77 ± 0.1	0.92
CFRC/Me	Carbon composite with melamine foam as the center layer	Sandwich structure	787.57	7.02 ± 0.2	0.1
CFRC/Cu/Me	Carbon composite integrated with Cu-coated PET and melamine foam in the center	Sandwich structure	828.83	8.1 ± 1.2	0.11
CFRC+Gr	Carbon fiber-reinforced composite with a graphite particles-mixed matrix	Two layers	651.24	0.98 ± 0.1	0.67
CFRC+Gr/Cu	Carbon fiber-reinforced composite with a graphite particles-mixed matrix and Cu-coated PET as the center layer	Sandwich structure	718.74	0.78 ± 0.06	0.92
CFRC+Gr/Me	Carbon fiber-reinforced composite with a graphite particles-mixed matrix and melamine foam as the center layer	Sandwich structure	735.07	7.73 ± 0.2	0.1
CFRC+Gr/Cu/Me	Carbon fiber-reinforced composite with a graphite particles-mixed matrix and Cu-coated PET and melamine foam in the center	Sandwich structure	824.05	7.62 ± 0.03	0.11

**Table 3:** Tensile test results of CFRC series compared with those of metal materials used for EV batteries.

Material	Tensile Strength (MPa)	Tensile Modules (GPa)	Specific Stength (MPa/(g/cm^3^))	Specific Modulus (GPa/(g/cm^3^))
CFRC	207.1 ± 11.4	3.8 ± 0.03	272.5	5
CFRC/Cu	197.6 ± 20.6	2.7 ± 0.3	182.1	2.9
CFRC/Me	18.3 ± 1.3	0.4 ± 0.03	182.7	4
CFRC/Cu/Me	17.6 ± 0.3	0.3 ± 0.03	160.2	2.7
CFRC+Gr	232.4 ± 7.7	4.2 ± 0.2	346.8	6.3
CFRC+Gr/Cu	295 ± 32.2	5.5 ± 0.3	320.7	6
CFRC+Gr/Me	17.8 ± 1.4	0.3 ± 0.01	178.2	3
CFRC+Gr/Cu/Me	14.1 ± 2.1	0.3 ± 0.05	128.2	2.7
6061-T6 aluminum alloy [34]	310–350	68–70	114.8–129.6	25.2–25.9
7075 High-strength aluminum alloy [35]	560–590	72–80	200–210.7	25.71–28.57
High-strength steel (HSS) [36]	500–1200	190–210	64.1–153.8	24.7–26.9
Ultra-high-strength steel [37]	1000–1750	190–220	128.2–224.4	24.36–28.21

## Data Availability

The original contributions presented in the study are included in the article, further inquiries can be directed to the corresponding author.

## References

[1] Goswami M., Kumar S., Siddiqui H., Chauhan V., Singh N., Sathish N., Ashiq M., Kumar S., Prabhansu, Kumar N. (2023). 22—Electric Vehicles: A Step toward Sustainability. Emerging Trends in Energy Storage Systems and Industrial Applications.

[2] Gupta P., Toksha B., Patel B., Rushiya Y., Das P., Rahaman M. (2022). Recent Developments and Research Avenues for Polymers in Electric Vehicles. Chem. Rec..

[3] Maske P., Chel P.A., Goyal P.K., Kaushik D.G. (2021). Sustainable Perspective of Electric Vehicles and Its Future Prospects. J. Sustain. Mater. Process. Manag..

[4] Kumar P., Srivastava K.N., Dhar A., Gautam A., De S., Dhar A., Gupta J.G., Pandey A. (2018). Role of Electric Vehicles in Future Road Transport. Sustainable Energy and Transportation: Technologies and Policy.

[5] Un-Noor F., Padmanaban S., Mihet-Popa L., Mollah M.N., Hossain E. (2017). A Comprehensive Study of Key Electric Vehicle (EV) Components, Technologies, Challenges, Impacts, and Future Direction of Development. Energies.

[6] Van Mierlo J., Berecibar M., El Baghdadi M., De Cauwer C., Messagie M., Coosemans T., Jacobs V.A., Hegazy O. (2021). Beyond the State of the Art of Electric Vehicles: A Fact-Based Paper of the Current and Prospective Electric Vehicle Technologies. World Electr. Veh. J..

[7] Rizzoni G., Ahmed Q., Arasu M., Oruganti P.S. (2019). Transformational Technologies Reshaping Transportation—An Academia Perspective.

[8] Buckingham R., Asset T., Atanassov P. (2021). Aluminum-Air Batteries: A Review of Alloys, Electrolytes and Design. J. Power Sources.

[9] Sadeghian A., Iqbal N. (2022). A Review on Dissimilar Laser Welding of Steel-Copper, Steel-Aluminum, Aluminum-Copper, and Steel-Nickel for Electric Vehicle Battery Manufacturing. Opt. Laser Technol..

[10] Sun G., Jia C., Zhang J., Yang W., Ma Z., Shao G., Qin X. (2019). Effectively Enhance High Voltage Stability of LiNi1/3Co1/3Mn1/3O2 Cathode Material with Excellent Energy Density via La_2_O_3_ Surface Modified. Ionics.

[11] Cherat N., Rijnkels M., Godthi V., Sharma H., P A., Bobba S. (2022). Thermoplastic Solution for Electric Vehicle Battery Protection.

[12] Choi C.-H., Cho J.-M., Kil Y., Yoon Y. (2013). Development of Polymer Composite Battery Pack Case for an Electric Vehicle.

[13] Maiti S., Islam M.R., Uddin M.A., Afroj S., Eichhorn S.J., Karim N. (2022). Sustainable Fiber-Reinforced Composites: A Review. Adv. Sustain. Syst..

[14] Harussani M.M., Sapuan S.M., Nadeem G., Rafin T., Kirubaanand W. (2022). Recent Applications of Carbon-Based Composites in Defence Industry: A Review. Def. Technol..

[15] Lim G.J.H., Chan K.K., Sutrisnoh N.A.A., Srinivasan M. (2022). Design of Structural Batteries: Carbon Fibers and Alternative Form Factors. Mater. Today Sustain..

[16] Wang Y., Hu S., Tunáková V., Niamlang S., Chvojka J., Venkataraman M., Militký J., Khan M.Z., Ali A. (2024). Carbon Felt from Acrylic Dust Bags as Flexible EMI Shielding Layer and Resistive Heater. J. Mater. Res. Technol..

[17] Sayam A., Rahman A.N.M.M., Rahman M.S., Smriti S.A., Ahmed F., Rabbi M.F., Hossain M., Faruque M.O. (2022). A Review on Carbon Fiber-Reinforced Hierarchical Composites: Mechanical Performance, Manufacturing Process, Structural Applications and Allied Challenges. Carbon Lett..

[18] Hu S., Wang D., Venkataraman M., Křemenáková D., Militký J., Yang K., Wang Y., Palanisamy S. (2023). Enhanced Electromagnetic Shielding of Lightweight Copper-Coated Nonwoven Laminate with Carbon Filament Reinforcement. J. Eng. Fibers Fabr..

[19] Silva F., Aragón M. Electromagnetic Interferences from Electric/Hybrid Vehicles. Proceedings of the 2011 XXXth URSI General Assembly and Scientific Symposium.

[20] Wang D., Hu S., Vecernik J., Kremenakova D., Militky J., Novotna J. (2024). Impact of Different Matrix Systems on Mechanical, Thermal, and Electrical Properties of Carbon Fiber Reinforced Epoxy Resin Matrix Composites. Polym. Compos..

[21] Santhosh M.S., Sasikumar R., Natarajan E. (2021). E-Glass/Phenolic Matrix/APP Laminate as a Potential Candidate for Battery Casing of e-Vehicle—Experimental Investigations. Mater. Res. Express.

[22] Malik M., Dincer I., Rosen M.A. (2016). Review on Use of Phase Change Materials in Battery Thermal Management for Electric and Hybrid Electric Vehicles. Int. J. Energy Res..

[23] Gryz K., Karpowicz J., Zradziński P. (2022). Complex Electromagnetic Issues Associated with the Use of Electric Vehicles in Urban Transportation. Sensors.

[24] Moreno-Torres P., Lafoz M., Blanco M., R.Arribas J., Moreno-Torres P., Lafoz M., Blanco M., Arribas J.R. (2016). Passenger Exposure to Magnetic Fields in Electric Vehicles. Modeling and Simulation for Electric Vehicle Applications.

[25] Aiello O. (2020). Electromagnetic Susceptibility of Battery Management Systems’ ICs for Electric Vehicles: Experimental Study. Electronics.

[26] Rao Z., Wang S. (2011). A Review of Power Battery Thermal Energy Management. Renew. Sustain. Energy Rev..

[27] Wu W., Wang S., Wu W., Chen K., Hong S., Lai Y. (2019). A Critical Review of Battery Thermal Performance and Liquid Based Battery Thermal Management. Energy Convers. Manag..

[28] Zhang X., Li Z., Luo L., Fan Y., Du Z. (2022). A Review on Thermal Management of Lithium-Ion Batteries for Electric Vehicles. Energy.

[29] Jaguemont J., Van Mierlo J. (2020). A Comprehensive Review of Future Thermal Management Systems for Battery-Electrified Vehicles. J. Energy Storage.

[30] (2017). Standard Test Methods for Apparent Density, Bulk Factor, and Pourability of Plastic Materials.

[31] (2018). Standard Test Method for Measuring the Electromagnetic Shielding Effectiveness of Planar Materials.

[32] (2014). Textiles—Physiological Effects—Measurement of Thermal and Water-Vapour Resistance under Steady-State Conditions (Sweating Guarded-Hotplate Test).

[33] (2017). Test Method for Tensile Properties of Polymer Matrix Composite Materials.

[34] Tan C., Said M.R., Chen W. The Tensile Strength Effects on Precipitation Heat Treatment of 6061-T6 Aluminum Alloy. Proceedings of the ASME 2009 International Design Engineering Technical Conferences and Computers and Information in Engineering Conference.

[35] Pei H.-W., Zhang S.-G., Guo H.-M., He W., Liu X.-B. (2022). Forming and Properties of 7075 Aluminum Alloy by Rheological Squeeze Casting with Transverse Mobile Injection Feed. Int. J. Adv. Manuf. Technol..

[36] Maraveas C., Fasoulakis Z.C., Tsavdaridis K.D. (2017). Mechanical Properties of High and Very High Steel at Elevated Temperatures and after Cooling Down. Fire Sci. Rev..

[37] Tian J., Jiang Z. (2021). Exploring New Strategies for Ultrahigh Strength Steel via Tailoring the Precipitates. Front. Mater..

[38] Zhang J., Wang J., Lv X. (2021). Simulation Study on the Influence of the Shielding Mechanism of the Battery Pack Shell on the Vehicle Radiation Emission.

[39] Mariscotti A. (2022). Harmonic and Supraharmonic Emissions of Plug-In Electric Vehicle Chargers. Smart Cities.

[40] Dini P., Saponara S., Colicelli A. (2023). Overview on Battery Charging Systems for Electric Vehicles. Electronics.

[41] Hu S., Wang D., Křemenáková D., Militký J. (2023). Washable and Breathable Ultrathin Copper-Coated Nonwoven Polyethylene Terephthalate (PET) Fabric with Chlorinated Poly-Para-Xylylene (Parylene-C) Encapsulation for Electromagnetic Interference Shielding Application. Text. Res. J..

[42] Wang D., Hu S., Kremenakova D., Militky J. (2022). The Electromagnetic Shielding Effectiveness of the Copper Plated Nonwoven Fabric and Its’ Related Comfort Properties. J. Eng. Fibers Fabr..

[43] Wang D., Hu S., Kremenakova D., Militky J. (2023). Evaluation of the Wearing Comfort Properties for Winter Used Electromagnetic Interference Shielding Sandwich Materials. J. Ind. Text..

